# Exposure to *p,p*′-DDE: A Risk Factor for Type 2 Diabetes

**DOI:** 10.1371/journal.pone.0007503

**Published:** 2009-10-19

**Authors:** Anna Rignell-Hydbom, Jonas Lidfeldt, Hannu Kiviranta, Panu Rantakokko, Göran Samsioe, Carl-David Agardh, Lars Rylander

**Affiliations:** 1 Division of Occupational and Environmental Medicine, Department of Laboratory Medicine, Lund University, University Hospital, Lund, Sweden; 2 Unit on Vascular Diabetic Complications, Department of Clinical Science, Lund University, University Hospital MAS, Malmö, Sweden; 3 National Institute for Health and Welfare (THL), Neulanen Research Centre, Kuopio, Finland; 4 Department of Obstetrics and Gynaecology, University Hospital Lund, Lund, Sweden; Ecole Normale Supérieure de Lyon, France

## Abstract

**Background:**

Persistent organic pollutants (POPs), such as PCBs, DDT and dioxins have in several cross-sectional studies shown strong associations with type 2 diabetes mellitus. Reversed causality can however not be excluded. The aim of this case-control study was to evaluate whether POPs concentration is a risk factor for type 2 diabetes.

**Methodology/Principal Findings:**

A case-control study was performed within a well-defined cohort of women, age 50–59 years, from the Southern part of Sweden. Biomarkers for POP exposure, 2,2′,4,4′,5,5′-hexachlorobiphenyl (CB-153) and 1,1-dichloro-2,2-bis (*p*-chlorophenyl)-ethylene (*p,p′*-DDE) were analyzed in stored serum samples, which were collected at the baseline examination when the cohort was established. For 107 out of the 371 cases, serum samples were stored at least three years before their type 2 diabetes was diagnosed. In this data set, CB-153 and *p,p′*-DDE were not associated with an increased risk to develop type 2 diabetes. However, when only the cases (n = 39) that were diagnosed more than six years after the baseline examination and their controls were studied, the women in the highest exposed quartile showed an increased risk to develop type 2 diabetes (OR of 1.6 [95% 0.61, 4.0] for CB-153 and 5.5 [95% CI 1.2, 25] for *p,p′*-DDE).

**Conclusions/Significance:**

The results from the present case-control study, including a follow-up design, confirms that *p,p′*-DDE exposure can be a risk factor for type 2 diabetes.

## Introduction

The incidence of type 2 diabetes is rapidly increasing world-wide [1]. The main factors identified as responsible for the disease are an aging population with a genetic predisposition for diabetes, change in lifestyle such as low physical activity, obesity and smoking. In addition, multiple reports corroborate the association between persistent organochlorine pollutants (POPs) such as polychlorinated biphenyls (PCBs), dioxins and dichloro-diphenyl-trichloroethane (DDT) and type 2 diabetes. Since the 1930s, PCBs and DDT have been manufactured and released into the environment. These pollutants are highly lipophilic, hence bio-accumulate in the food chain and due to their long half lives they are still detected in humans even though they were banned in most countries in the 1970s and 1980s [2]. Concentrations of most of these pollutants have been diminishing in the environment, the food chain and the human body over recent decades, in most Western countries. However, there are subgroups in the general population that still show an elevated body burden due to dietary habits and current or past exposures. In studies with a cross-sectional design surprisingly strong associations have been shown between serum concentrations of POPs and type 2 diabetes [3]–[14]. If these associations reflect a true effect of environmental exposures on the incidence of diabetes, then this is the largest public health effect observed for POPs so far. However, the majority of recent studies were cross-sectional and a direct cause has so far not been shown, thus follow-up studies are needed.

Altered lipid metabolism [15], altered glucose transport [16], [17] and alterations in the insulin signaling pathway [18] are potential mechanisms that might be involved in the association between POPs and diabetes. Diabetes in itself is also known to cause a dysregulation of fat metabolism, which in turn might influence the distribution and elimination of POPs. Information from a recent study of insulin sensitivity and POPs in pregnant women also shows an association within the non-pathologic range of insulin sensitivity [19].

A recent commentary by Jones and colleagues stressed the importance of new clinical, toxicological, and epidemiological studies, in particular those that integrate several levels of evidence over a long period [20]. The aim of the present case-control study, performed within a well-defined cohort of women from the Southern part of Sweden, was to elucidate to what extent POP exposure may have contributed to the development of type 2 diabetes. We made the analysis of two biomarkers for POP exposure, 2,2′,4,4′,5,5′-hexachlorobiphenyl (CB-153) and 1,1-dichloro-2,2-bis (*p*-chlorophenyl)-ethylene (*p,p′*-DDE), in serum collected before the cases had type 2 diabetes diagnosed.

## Materials and Methods

### Study population and design

Between December 1995 and February 2000 a generic survey based on a questionnaire, physical examinations, and laboratory assessments were completed on 6 917 women (corresponding to 64% participating rate) aged 50–59 years and living in the five municipalities in the Lund area, located in Southern Sweden (the Women's Health In the Lund Area cohort - WHILA). A detailed description of the WHILA cohort has been given elsewhere [21]. In brief, the women were discriminated as positive or negative to each one of eight variables linked to the metabolic syndrome (hyperglucemia, dyslipidemia, obesity and hypertensive disorders). Women with the metabolic syndrome features (positive, n = 3144) underwent a baseline Oral Glucose Tolerance Test (OGTT), one to four weeks later. By linkage with the Swedish in-patient and out-patient registers, women from the WHILA cohort who had developed type 2 diabetes before 31 December 2006, were identified. A randomly selected subgroup (n = 221) of women without the metabolic syndrome features, also underwent OGTT, and the results corroborated the very low prevalence of previously unknown diabetes among women outside the group. Women with previously confirmed diabetes (n = 139) were excluded from further studies. In total, 410 women were diagnosed with type 2 diabetes after the baseline examination. A case-control study was performed within the WHILA cohort. Blood samples were obtained from all participating women at the baseline examination and were stored at −70°C until the present POP analyses were run. The study was performed in accordance with the Declaration of Helsinki and approved by the Research Ethics Committee at Lund University. All participants accepted with verbal informed consents.

### Cases

Out of 410 incident diabetic women (cases) 39 were not eligible for the current study due to lack of serum samples. Background characteristics for the remaining 371 cases are presented in Table 1. Fifty-six percent were diagnosed with type 2 diabetes within one year after baseline examination.

**Table 1:** Background characteristics for 371 women from the Southern part of Sweden who were diagnosed for type 2 diabetes after the baseline investigation (cases) and a corresponding number of matched control women.

	Controls (n = 371)	Cases (n = 371)
Variables	*Median (Min, Max)*	*Median (Min, Max)*
Calendar-year at baseline^a^	1998 (1995, 2000)	1998 (1995, 2000)
Age at baseline (years)^a^	57.6 (50.7, 63.8)	57.3 (51.1, 63.8)
BMI at baseline (kg/m^2^)^a^	28.5 (18.2, 43.8)	28.3 (17.9, 47.0)
BMI at 25 years of age (kg/m^2^)	21.7 (15.8, 33.7)	21.4 (15.4, 34.6)
Time between baseline and T2DM diagnosis (years)		0.23 (0.01, 10.5)
	*Percent*	*Percent*
Family history of T2DM^b^	17	18
Born in Sweden	91	91
Education		
Compulsory school	29	32
Senior high school	44	45
University	27	23
Smoking history at baseline		
Ex smoker	20	22
Current smoker	12	16
Moderate/High alcohol intake^c^	9	16
Hormone replacement therapy at baseline	30	34
Low leisure time exercise^d^	64	66
Low physical activity at work^e^	33	35

a Matching variable.

b First-degree relatives.

c More than 84 gram alcohol per week.

d Less than one hour of strenuous training session per week.

e Mostly sedentary work.

### Controls

For each case, one control was randomly selected from the WHILA cohort, matched for age, calendar-year, body mass index (BMI), and according to positive or negative selection criteria for OGTT at the baseline examination, i.e. presence or not of any features of the metabolic syndrome.

### Biomarkers of exposure

In the present study CB-153 has been used as a biomarker for PCB exposure. CB-153 correlates very well (r = 0.98) with both total PCB concentrations in plasma and serum from Swedish subjects [22], [23] and with 2,3,7,8-tetrachlorodibenzo-*p*-dioxin (TCDD) equivalent (TEQ) in plasma from PCB (r = 0.89). Moreover, the major DDT metabolite *p,p′*-DDE which has anti-androgenic effect and is found in relatively high serum concentrations in adult Swedish people, has also been used [24].

### Chemical analyses

All 742 small (200 µl) serum samples were shipped frozen to the Laboratory of Chemistry (National Public Health Institute) in Kuopio in 21^st^ January 2008, where the levels of two individual POPs; *p,p′*-DDE and CB 153 were measured as pg/ml of serum. The chemical analyses have recently been described in detail [25]. Briefly, each sample of serum, 200 µl, was pipetted to a pre-cleaned 8 ml glass test tube, and spiked with ^13^C-labelled *p,p′*-DDE and CB-153 internal standards. Ethanol (p.a. purity) was added to precipitate proteins and equilibrate the internal standards during the 5 min period in ultrasonic bath. For extraction of analytes, 2.0 ml of hexane (p.a. purity) was added to sample and the sample tube was vortexed for 10 min at 2000 rpm with an automatic shaker. The sample was cleaned up by adding 1.0 ml of 15% sulphuric acid impregnated silica and continuing the vortex for 5 minutes more. The sample was centrifuged for 2 min in 3500 rpm to form a solid precipitate. The clear hexane fraction on top was poured into another test tube, evaporated to a small volume, transferred to autosampler vial, and a recovery standard (^13^C CB-128) was added. The final volume of the sample was adjusted to 200 µl of toluene.

Laboratory reagent blanks and control serum samples were pipetted and prepared using test tubes from the same batch as real samples. No signs of contamination or losses by adsorption were observed in these quality control samples.

The quantification was performed by high resolution mass spectrometer (Micromass Ultima, Waters) with electron impact ionisation and selective ion recording with a resolution of 10 000. Two most intensive ions of each analyte or internal standard were monitored. Mass spectrometer was equipped with a HP 6890 (Hewlett Packard) gas chromatograph. A fused silica capillary column, DB-5 MS (length, 30 m; ID, 0.25 mm; film thickness, 0.25 µm) was used to separate the analytes. Two µl was injected into a split-splitless injector kept at 280°C. The temperature program for the gas chromatograph was: 140°C (2 min), 20°C/min to 190°C (0 min), 8°C/min to 260°C (0 min), 40°C/min to 300°C (4 min).

Limits of detection and limits of quantification were 4.3 and 11.5 pg/ml for *p,p′*-DDE, and 3.1 pg/ml and 8.2 pg/ml for CB-153, respectively. These were significantly lower than the lowest measured concentrations in the cohort (63 pg/ml for *p,p′*-DDE and 88 pg/ml for CB-153).

The Laboratory of Chemistry is an accredited testing laboratory (T077). The scope of accreditation covers POPs from serum samples.

### Statistical analyses

The association between POP exposure and risk of developing type 2 diabetes was evaluated by conditional logistic regression (EGRET), given odds ratios (OR) as the risk measure with 95% confidence intervals (CI). The exposure variables (CB-153 and *p,p′*-DDE) were analyzed as continuous variables as well as categorized into quartiles and tertiles, respectively, based on the distributions among all controls. Women with serum concentrations in the highest quartile (or tertile) were considered as exposed. In addition, separate analyses were performed for the set of cases and controls where the cases had their type 2 diabetes diagnosed at least one, three, five and seven years after the base-line examination, respectively. The correlation between the serum concentrations of CB-153 and *p,p′*-DDE was relatively high (r_s_ = 0.66), and we did therefore not include these exposure values simultaneously in the models.

The similarities between cases and controls regarding age at primary screening, calendar-year and body mass index, confirm that the matching was successfully performed (Table 1). We did analyse our case-control data with conditional logistic regressions and did accordingly, by definition, adjust for these variables. Other background characteristics and potential risk factors for type 2 diabetes, such as heredity, country of birth, education, smoking history, alcohol intake, hormone replacement therapy, and physical activity were very similar among cases and controls (Table 1). None of these latter variables had any strong association with POP exposure levels and we did therefore not include them as potential confounders in the models.

## Results

The mean concentrations of CB-153 and *p,p′*-DDE among all cases were very similar to all controls (Table 2). For the set of cases and controls where the cases had type 2 diabetes diagnosed at least seven years after the baseline examination (n = 39), the cases had a 22% higher mean concentration of CB-153 (1560 and 1280 pg/mL) and a 46% higher mean concentration of *p,p′*-DDE (5680 and 3890 pg/mL) compared with controls.

**Table 2:** Serum concentrations (pg/mL) of CB-153 and *p,p′*-DDE among women from the Southern part of Sweden who were diagnosed with type 2 diabetes after the baseline investigation (cases) and a corresponding number of matched control women.

	All		<1 year		>3 years		>7 years	
	Controls (n = 371)	Cases (n = 371)	Controls (n = 208)	Cases (n = 208)	Controls (n = 107)	Cases (n = 107)	Controls (n = 39)	Cases (n = 39)
CB-153								
Mean (sd)	1470 (740)	1440 (780)	1510 (770)	1480 (840)	1440 (740)	1450 (710)	1280 (730)	1560 (890)
Median (Min, Max)	1340 (88, 6330)	1270 (250, 7280)	1400 (88, 6330)	1260 (250, 7280)	1290 (190, 5134)	1340 (310, 4160)	1100 (190, 3360)	1360 (380, 4160)
Fraction >1790^a^	25	25	27	26	22	28	23	33
*p,p′*-DDE								
Mean (sd)	3760 (3550)	4110 (4460)	3770 (3880)	3830 (4120)	3930 (3160)	5140 (5550)	3890 (3770)	5680 (6160)
Median (Min, Max)	2890 (63, 34860)	2890 (160, 39770)	2750 (63, 34860)	2780 (160, 39770)	3050 (500, 17750)	3370 (260, 29190)	2890 (530, 17750)	3610 (280, 29190)
Fraction >4600^a^	25	26	24	22	27	35	20	44

Figures are also given for the set of cases and controls were the cases had T2DM diagnosed <1, >3, or >7 years after the baseline investigation.

a Corresponding to the 75^th^ percentile among all controls.

When all individuals were included in the analyses, the women in the highest exposure quartile showed no increased risk to develop type 2 diabetes as compared to women in the three lower quartiles, irrespectively if investigating the concentrations for CB-153 or *p,p′*-DDE (OR 0.99 and 1.1, respectively, Table 3). The corresponding ORs increased gradually, i.e. the longer time that had passed between the baseline examination and the time to diagnose. If the cases were diagnosed at least seven years after the baseline examination, OR of 1.6 (95% 0.61, 4.0) for CB-153 and 5.5 (95% CI 1.2, 25) for *p,p′*-DDE were obtained. The patterns observed in Table 3 were very much the same if we replaced the highest quartiles with the highest tertiles as the exposed category (data not shown). Moreover, when we used continuous exposure variables, the picture was similar, i.e., the higher effect estimates, the longer time that had passed between the baseline examination and the time to diagnose (data not shown).

**Table 3:** Odds ratios (OR) with 95% confidence intervals (CI) obtained from conditional logistic regressions.

	OR	95% CI
CB-153 (pg/mL)		
*>1790 vs ≤1790 (ref)* a		
All (371 sets^b^)	0.99	0.71–1.4
<1year (208 sets^b^)	0.91	0.59–1.4
>1year (163 sets^b^)	1.1	0.66–1.9
>3 years (107 sets^b^)	1.4	0.72–2.6
>5 years (74 sets^b^)	1.4	0.67–3.1
>7 years (39 sets^b^)	1.6	0.61–4.0
*p,p′*-DDE (pg/mL)		
*>4600 vs ≤4600 (ref)* a		
All (371 sets^b^)	1.1	0.76–1.5
<1year (208 sets^b^)	0.90	0.57–1.4
>1year (163 sets^b^)	1.3	0.78–2.2
>3 years (107 sets^b^)	1.5	0.80–2.8
>5 years (74 sets^b^)	2.5	0.97–6.4
>7 years (39 sets^b^)	5.5	1.2–25

Figures are given when all women were included in the analyses, as well as separately for the set of cases and controls were the cases had type 2 diabetes diagnosed <1, >1, >3, >5 or >7 years after the baseline investigation.

a The cut-off level corresponding to the 75^th^ percentile among all women.

b n Sets = n cases+n controls.

## Discussion

Among women from the general population, the present study indicate that high serum concentrations of *p,p′*-DDE is a strong risk factor for developing type 2 diabetes later in life. A five-fold statistically significant increased risk was observed among the individuals with the longest follow-up. This finding is in accordance with the results from previous cross-sectional studies [3]–[9], [12], [26]. Although less pronounced, similar pattern was observed for CB-153. Thus, our data support that POP exposure can be a risk factor for type 2 diabetes.

The present study has some possible limitations. The majority (56%) of the cases were diagnosed within one year after baseline examination. At baseline examination some of the women with hypertensive disorders or dyslipidemia, that required pharamcological treatment, were referred to their general practitioner (GP) and these contacts might have led to increased awareness of the risk to develop diabetes and therefore the women were diagnosed shortly after baseline examination. However, the majority of the women with some metabolic features at baseline examination, were only given life style consultation regarding exercise, smoking, diet and alcohol use. We can only speculate, but it is reasonable to believe that after being aware of how their life style might trigger the risk to develop diabetes, the women in this group contacted their GPs for a new examination. This has implications for the statistical power regarding the long term follow-up of the study. Although the strong associations observed in our study did reach statistical significance, it has to be confirmed in larger follow-up studies. Further, in the original WHILA study, 36% did not participate. Possible differences between participants and non-participants have previously been thoroughly analyzed [21] and in the present study we believe that the relatively high fraction of non-participants in the original cohort study is of minor importance. The reason for this statement is that women from the general population are not aware of their body burden of POPs.

Due to the limited amount of serum available from the biobank, the chemical analyses was restricted to CB-153 and *p,p*′-DDE and we had no possibility to lipid adjust our samples. Although there are no experimental data supporting that di-ortho PCB congeners such as CB-153, will have a diabetogenic effect by themselves, we know from other studies that CB-153 serves as a good proxy biomarker for total PCB as well as for TCDD TEQ [23]. *p,p*′-DDE was selected due to its anti-androgenic properties and because it is still found in relatively high serum concentrations among adult Swedish people [24]. Regarding the lack of lipid adjusted exposure measurements, we do not see this as a major problem due to the very strong correlations (r>0.90) found between fresh and lipid adjusted samples [22]. Moreover, concentrations of serum triglycerides, at baseline examination, did not differ between cases and controls. However, we can not exclude that other factors created some statistical noise, thereby biasing the results toward the null hypothesis, which means that we underestimate the true extent of an effect.

The major strength of the study was the access to stored serum samples from women participating in the WHILA study, which was designed for investigating risk factors for developing type 2 diabetes and therefore includes information on well-known risk factors. The cohort is well-defined and more than 90% of the women in the present study were born in Sweden and had Caucasian ethnicity. In addition, the criteria for type 2 diabetes was well described [27] and the cases had their diagnosis given after a medical examination and were not self reported. Another important aspect of the present study is that for 107 cases the concentrations of CB-153 and *p,p′*-DDE were measured at least three years before type 2 diabetes was diagnosed. This means that the present study is one of very few studies with a prospective design, and to the best of our knowledge, has the largest numbers of cases. A very recent prospective study from the US, with somewhat lower concentrations of *p,p′*-DDE, did also show strong associations between *p,p′*-DDE serum levels and the risk of developing type 2 diabetes [13]. The study did, however, only include nine incident female cases.

Levels of PCBs and *p,p′*-DDE have been declining in the general population in Sweden over the past decades whereas the incidence and prevalence of type 2 diabetes has increased quite dramatically, as for most industrialized countries, during the same time period [1], [28].

Since both *p,p′*-DDE and CB-153 have a long half life (five to ten years), we believe that long term exposure, over a period of several decades, at least in part reflect the concentration present. [29].

Recent studies have shown strong associations between POP exposure levels in the general population and type 2 diabetes. This might look peculiar, in view of the currently decreasing levels of POPs present in the environment. There are however some possible explanations to this circumstance. First, obesity which undoubtedly is a well known risk factor to type 2 diabetes is increasing in the western world [30]. In a study by Lee and co-workers [26] an association between obesity and diabetes became stronger as serum concentrations of POPs increased. However, obesity was not associated with diabetes among individuals with very low concentrations of POPs, in whom the prevalence of diabetes itself was quite low. This could imply that POPs stored in adipose tissue may play a role in the current epidemic of type 2 diabetes.

Secondly, endocrine disrupters (EDs) alter normal hormonal regulation and they may be naturally occurring or environmental contaminants. Most of the “old” POPs (like PCBs and DDT) and the “new” such as brominated flame-retardants, polyfluorinated compounds, and other non-persistent contaminants such as phthalates and Bispheol A, have endocrine disrupting properties [31]–[33]. We do not know whether mixtures of different EDs (the so-called cocktail effect) are synergistic or additive. The women in our study were postmenopausal, i.e. with decreasing internal estrogen levels and to some extent decreasing testosterone levels. Some women might be more susceptible to these pollutants than others, and a body burden of POPs, for a long period, might cause a disruption of hormonal balance and disrupt normal metabolism of glucose and lipids. Recent reports indicate that EDs have the potential to stimulate the lipid accumulation in target cells, such as adipocytes and hepatocytes, related to obesity and metabolic syndrome, and the accumulation may cause dysfunction in these cells resulting in induction of the metabolic syndrome [34]. Furthermore, EDs might act by binding to sites that rapidly activate different signalling cascades which affect the normal physiology of the endocrine pancreas, altering the regulation of glucose and lipid metabolism [35].

This taken together makes it reasonable to believe that p,p′-DDE and CB-153 might trigger development of type 2 diabetes.

A major part of the women in the current study were teenagers when the exposures to CB-153 and *p,p′*-DDE were at the highest levels in Sweden. The most critical exposure window for the risk of developing type 2 diabetes is not known. One might speculate that exposure during puberty is of importance. In the present study we do not know whether the exposure levels measured is correlated with the levels earlier in life. To clarify this issue, we do recommend future studies to focus on different exposure windows.

In conclusion, it has been suggested that environmental pollutants might be a part of the explanation for increased incidence of type 2 diabetes. Several cross-sectional studies have indicated strong associations between POP exposure levels and type 2 diabetes, but the question of reversed causality remained unanswered. The results from the present case-control study, with a follow-up design, confirms an association between *p,p′*-DDE exposure and type 2 diabetes.
